# Developing Machine Learning Algorithms for the Prediction of Early Death in Elderly Cancer Patients: Usability Study

**DOI:** 10.2196/12163

**Published:** 2019-09-26

**Authors:** Gabrielle Ribeiro Sena, Tiago Pessoa Ferreira Lima, Maria Julia Gonçalves Mello, Luiz Claudio Santos Thuler, Jurema Telles Oliveira Lima

**Affiliations:** 1 Department of Geriatric Oncology Instituto de Medicina Integral Prof Fernando Figueira Recife Brazil; 2 Instituto Federal de Pernambuco - IFPE Department os Computational Science Recife Brazil; 3 Research Center Instituto Nacional do Cancer - INCA Rio de Janeiro Brazil

**Keywords:** geriatric assessment, aged, machine learning, medical oncology, death

## Abstract

**Background:**

The importance of classifying cancer patients into high- or low-risk groups has led many research teams, from the biomedical and bioinformatics fields, to study the application of machine learning (ML) algorithms. The International Society of Geriatric Oncology recommends the use of the comprehensive geriatric assessment (CGA), a multidisciplinary tool to evaluate health domains, for the follow-up of elderly cancer patients. However, no applications of ML have been proposed using CGA to classify elderly cancer patients.

**Objective:**

The aim of this study was to propose and develop predictive models, using ML and CGA, to estimate the risk of early death in elderly cancer patients.

**Methods:**

The ability of ML algorithms to predict early mortality in a cohort involving 608 elderly cancer patients was evaluated. The CGA was conducted during admission by a multidisciplinary team and included the following questionnaires: mini-mental state examination (MMSE), geriatric depression scale-short form, international physical activity questionnaire-short form, timed up and go, Katz index of independence in activities of daily living, Charlson comorbidity index, Karnofsky performance scale (KPS), polypharmacy, and mini nutritional assessment-short form (MNA-SF). The 10-fold cross-validation algorithm was used to evaluate all possible combinations of these questionnaires to estimate the risk of early death, considered when occurring within 6 months of diagnosis, in a variety of ML classifiers, including Naive Bayes (NB), decision tree algorithm J48 (J48), and multilayer perceptron (MLP). On each fold of evaluation, tiebreaking is handled by choosing the smallest set of questionnaires.

**Results:**

It was possible to select CGA questionnaire subsets with high predictive capacity for early death, which were either statistically similar (NB) or higher (J48 and MLP) when compared with the use of all questionnaires investigated. These results show that CGA questionnaire selection can improve accuracy rates and decrease the time spent to evaluate elderly cancer patients.

**Conclusions:**

A simplified predictive model aiming to estimate the risk of early death in elderly cancer patients is proposed herein, minimally composed by the MNA-SF and KPS. We strongly recommend that these questionnaires be incorporated into regular geriatric assessment of older patients with cancer.

## Introduction

### Background

Aging is a complex and personal, cumulative, and irreversible phenomenon that goes well beyond chronological age [1]. It involves several biological events associated with a great variety of molecular and cellular damage, leading to the gradual loss of physiological and immunological reserves and a greater risk for neoplasia-related death [1,2]. Assuming that the elderly population is heterogeneous, this population must be considered not only concerning their chronological age. Thus, an objective analysis of their living conditions as well as aspects related to oncological disease and its therapy is also required [3].

The International Society of Geriatric Oncology has recommended the use of the Comprehensive Geriatric Assessment (CGA) for the evaluation and follow-up of elderly cancer patients [4]. The CGA is a multidisciplinary tool that uses validated instruments to evaluate several elderly health condition domains, such as functional, cognitive, psychological, social, clinical, and nutritional aspects, as well as comorbidities and the use of medication, among others [5,6]. It is also strongly recommended by the geriatrics and gerontology fields in general because it is, in a complex and heterogeneous context, an objective, measurable, and reproducible form of evaluation, adding possibilities to standard clinical laboratory evaluations [7,8]. However, there is no consensus about what and how many instruments should be used. Employing CGA in practice, however, has become a huge challenge, and owing to its complexity and time spent in its application, it is often underutilized by oncologists and not judged as a completely satisfactory solution in practice, which has served as a stimulus for the construction of simpler tools that have the power to predict outcomes and guide clinical decisions [5,9].

The accurate prediction of a disease outcome is one of the most interesting and challenging tasks for physicians. As a result, a growing trend was noted in the studies published during the past years that applied machine learning (ML) algorithms for modeling cancer survival. This type of algorithms can discover and identify patterns and relationships between them, from complex databases, while they are able to effectively predict future outcomes of a cancer type [10]. On the basis of the study by Kourou et al [11], the accuracy of cancer prediction outcome has significantly improved by 15% to 20% in the previous years, with the application of ML techniques.

A study combining data from 4 cohorts involving the elderly, 1 including elderly people with neoplasms, proposed to explore the performance of various ML classifiers (Naive Bayes [NB], k-nearest neighbors, artificial neural networks, random forest, and logistic regression) regarding death prediction in 6 months [12]. Another study used ML to predict mortality of patients in 3 to 12 months and to identify patients who could benefit from palliative care [13]. However, no ML application has been proposed using CGA to classify elderly cancer patients.

### Objectives

Thus, the primary aim of this study was to propose and develop predictive models, using ML and CGA, to estimate the risk of early death in elderly cancer patients. The secondary aims were to optimize the CGA through the selection of the most appropriate instruments.

## Methods

### Comprehensive Geriatric Assessment

The ability of ML techniques to predict early mortality in a heterogeneous cohort was tested in 608 elderly cancer patients (aged over 60 years), admitted to the oncogeriatrics sector of the Instituto de Medicina Integral Prof. Fernando Figueira - IMIP, from January 2015 to July 2016. The IMIP is a teaching hospital and cancer center located in Recife, Pernambuco, Brazil. On admission to the cohort database, the patients were evaluated by CGA questionnaires presented in Table 1. The questionnaires were collected by a multiprofessional team, comprising a clinical oncologist, a geriatrician, a physiotherapist, a physical educator, a speech therapist, an occupational therapist, and a nutritionist. The project was approved by the IMIP Ethics Committee on Human Research on June 30, 2016, under number 58298316.5.0000.5201.

**Table 1:** Questionnaires/features to evaluate elderly health condition domains in Comprehensive Geriatric Assessment.

Questionnaire/feature	Perspective	Range/cutoff
Charlson comorbidity index [14]	A prospective method for classifying comorbid conditions that might alter the risk of mortality	0 to 37 points, with an increase of up to 5 points per age range
Geriatric depression scale-short form [15]	A self-report measure of depression in older adults. Users respond in a yes/no format	score 0 to ≤5 is normal; score >5 is depression
International physical activity questionnaire-short form [16]	A set of questionnaires to obtain international comparative data on physical activity	0 is sedentary, 1 is insufficiently active, 2 is active, 3 is active, and 4 is very active
Karnofsky performance scale [17]	Used to quantify patients’ general well-being and activities of daily life	0 to 100, the lower the score, the worse the survival for most serious illnesses
Katz index of independence in activities of daily living [18]	Was developed to study results of treatment and prognosis in the elderly and chronically ill. Grades of the index summarize overall performance in bathing, dressing, going to toilet, transferring, continence, and feeding	0 to 6, high score means patient is independent and low score means patient is very dependent
Mini-mental state examination [19]	A method for grading the cognitive state of patients for the clinician	0 to 30, the lower the score rate, the worse the cognitive impairment
Mini nutritional assessment-short form [20]	A screening tool used to identify older adults who are malnourished or at risk of malnutrition. Comprises 6 questions on food intake, weight loss, mobility, psychological stress or acute disease, presence of dementia or depression, and body mass index	0 to 14, scores of 12-14 are considered normal nutritional status; 8-11 indicate at risk of malnutrition; and 0-7 indicate malnutrition
Polypharmacy [21]	Refers to the regular use of a greater number of medicines (5 or more drugs)	0 is no; 1 is yes
Timed up and go [22]	The patient is observed and timed while he rises from an arm chair, walks 3 m, turns, walks back, and sits down again	0 is low risk of falling (less than 20 seconds), 1 is average risk of falling (20-29 seconds), and 2 is high risk of falling (30 seconds or more)

### Preprocess of Database

The first step was to remove patients presenting redundancies and/or incomplete questionnaires/features. A total of 543 patients remained after that. Data normalization technique for equalizing the range of features, usually employed in the database before feature selection and learning phase, is of important concern in pattern recognition and computer-aided diagnosis [23]. The most common normalization method used during data transformation is the min-max (where the features are mapped into a predefined range, varying from 0 or −1 to 1). The main advantage of min-max normalization is that it preserves the relationships among the original data values [24]. In this work, all features were normalized in a (0,1) interval, calculated as in equation, where *v′* is the value normalized, *v* is the original value, *v_min_* is the minimum value of corresponding feature, and *v_max_* is the maximum value of corresponding feature: *v*’=(*v*−*v*_min_)/(*v*_max_−*v*_min_).

### Predictive Models

Predictive modeling is the general concept of building a model that can make predictions. Typically, such a model includes an ML algorithm that learns certain properties from a database to make those predictions. We have presented below a brief summary of the commonly used supervised learning algorithms:

- Decision tree J48 (J48) [25]: They are tree-like graphs, where the nodes in the graph test certain conditions on a set of features and the branches split the decision toward the leaf nodes. The leaves represent the lowest level in the graph and determine the class labels.
- Multilayer perceptron (MLP) [26]: They are graph-like classifiers that mimic the structure of a human or animal brain where the interconnected nodes represent the neurons.
- Naïve Bayes (NB) [27]: They are based on a statistical model (ie, Bayes theorem, calculating posterior probabilities based on the prior probability and the so-called likelihood).

**Figure 1:**
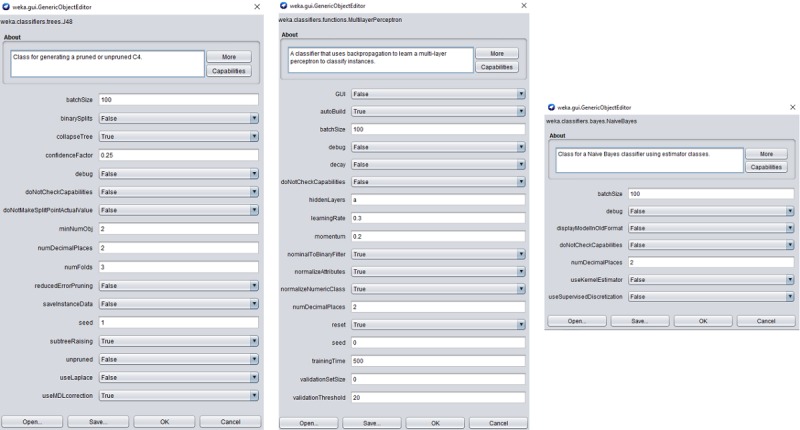
Parameters used in Decision Tree (J48), Multilayer perceptron, and Naive Bayes algorithms.

The purpose of this work was not to introduce the highest accuracy prediction model. The goal was to designate the most relevant questionnaires to evaluate elderly health condition domains in CGA. Therefore, in the experiments, we always used the same configuration with the default parameter values in Weka (Waikato Environment of Knowledge Analysis) from The University of Waikato, version 3.8.3. The advantage of using default parameters is that it does not introduce optimistic bias by tuning the parameter to maximize performance on the test data. Figure 1 shows more details about the values used in each predictive model.

### K-Fold Cross-Validation

Cross-validation (CV) [28] is one of the most widely used methods to assess the generalizability of predictive models [29] and is subject to ongoing active research [30]. K-fold CV comprises dividing the database into K parts (folds) of equal sizes. For this study, a 10-fold CV is used, and each part is held out in turn and the predictive model (J48, MLP, or NB) is trained on the remaining nine-tenths; then, its error rate is calculated on the holdout set. Thus, the learning procedure is executed a total of K times on different training sets (each of which have much in common). Finally, the K error estimates are averaged to yield an overall error estimate. In this work, the folds are made by preserving the percentage of samples for each class.

### Imbalanced Learn

The learning procedure and the subsequent prediction of predictive models can be affected by the problem of imbalanced database [31]. The balancing issue corresponds to the difference in the number of samples in the different classes. The resulting database presented 92 deaths within 6 months of admission to the service and 451 patients alive at the end of that period. All deaths were attributed to cancer (treatment complications or disease progression). With a greater imbalanced ratio, the decision function favors the class with the largest number of samples, usually referred as the majority class. The way to fight this issue was to generate new training sets on 10-fold CV by random sampling so that the proportion between classes remained at one-to-one.

### Metrics

The area under receiver operating characteristics curve, or simply area under curve (AUC), has recently been proposed as an alternative single-number measure for evaluating the generalization of learning algorithms [32]. This measure is far better than classification accuracies when the 2 classes are unbalanced and the cost of misclassification is unspecified [33]. An area of 1.0 represents a model that made all predictions perfectly, and an area of 0.5 represents a model as good as random. AUC can be broken down into sensitivity and specificity:

- Sensitivity is the true positive rate, and for this study, it is the percentage of patients with early death that are predicted correctly.
- Specificity is also called the true negative rate, for example, the percentage of patients without early death that are predicted correctly.

## Results

### Evaluating All Possible Combinations of Comprehensive Geriatric Assessment Questionnaires

**Figure 2:**
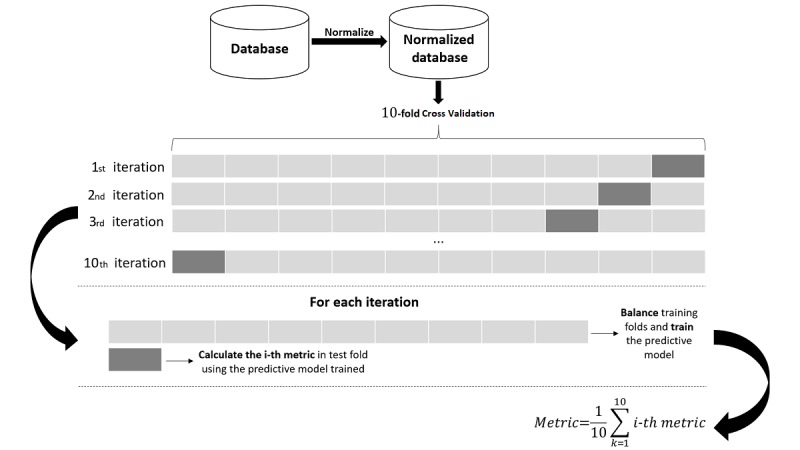
Flowchart of methodology.

Feature selection is an important and frequently used technique for dimension reduction by removing irrelevant and/or redundant information from the database to obtain an optimal feature subset. A 10-fold CV was used to evaluate all possible combinations of CGA questionnaires, presented in Table 2, to estimate the risk of early death in elderly cancer patients. Thus, in each fold, the combination of questionnaires with highest AUC is selected. The same folds are applied to all 511 combinations. Tiebreaking is handled by choosing the smallest set of questionnaires. The occurrence of questionnaires selected on the 10-fold CV, using predictive models, is presented in Table 2. In Figure 2, the flowchart of our methodology is shown.

**Table 2:** Occurrence of the Comprehensive Geriatric Assessment questionnaires in the 10-folds using decision tree (J48), multilayer perceptron, and Naive Bayes.

Model	Charlson comorbidity Index	Geriatric depression scale-short form	International physical activity question naire-short form	Katz index of independence in activities of daily living	Karnofsky performance scale	Mini-mental state examination	Mini nutritional assessment-short form	Polypharmacy	Timed up and go
Decision tree	6	4	0	4	1	1	10	2	2
Multilayer perceptron	0	0	0	0	10	1	10	0	0
Naive Bayes	9	6	6	0	10	10	10	2	0

### Evaluating combinations of occurrences

Tables 3-5 show the sensibility, specificity, and AUC values expressed as mean (SD) on the 10-fold CV for the NB, J48, and MLP. The subsets of CGA questionnaires, presented in these tables, consider the occurrences of Table 2. The subset of questionnaires with occurrence ≥0, for example, uses all set of CGA questionnaires, as it considers all occurrences greater than or equal to 0. The other subsets use the same logic and are detailed in the footnotes under the tables. In each metric, according to the paired *t* test, the *P* value is calculated considering the subset of questionnaires with occurrence ≥0. The experimental results demonstrate that the feature selection can discard questionnaires and finally find out subsets that reduce the dimensionality of data to make the predictive models more efficient and the results more accurate. Thus, a simplified predictive model aiming to estimate the risk of early death in elderly cancer patients is proposed herein, minimally composed by the Mini Nutritional Assessment-Short Form (MNA-SF), accompanied or not by the Karnofsky performance scale (KPS) and/or the Mini-Mental State Examination.

**Table 3:** Metrics considering Comprehensive Geriatric Assessment questionnaire subsets on Naive Bayes classifier.

Metric	Subsets of questionnaires with occurrence						
	≥0 occurrences^a^	≥6 occurrences^b^		≥9 occurrences^c^		10 occurrences^d^	
	Mean (SD)	Mean (SD)	*P* value	Mean (SD)	*P* value	Mean (SD)	*P* value
Sensibility	81.61 (4.62)	78.50 (6.3)	.02	80.28 (6.79)	.22	78.51 (5.00)	.003
Specificity	65.89 (14.72)	76.89 (12.48)	.002	71.45 (13.35)	.03	72.56 (12.31)	.01
AUC^e^	82.43 (6.35)	83.35 (6.9)	.16	83.31 (6.8)	.17	82.82 (6.78)	.37

^a^≥0 occurrences: All comprehensive geriatric assessments (Charlson comorbidity index, geriatric depression scale-short form, international physical activity questionnaire-short form, Katz index of independence in activities of daily living, Karnofsky performance scale, mini-mental state examination, mini nutritional assessment-short form, polypharmacy, and timed up and go).

^b^≥6 occurrences: Charlson comorbidity index, geriatric depression scale-short form, international physical activity questionnaire-short form, Karnofsky performance scale, mini-mental state examination, and mini nutritional assessment-short form.

^c^≥9 occurrences: Charlson comorbidity index, Karnofsky performance scale, mini-mental state examination, and mini nutritional assessment-short form.

^d^10 occurrences: Karnofsky performance scale, mini-mental state examination, and mini nutritional assessment-short form.

^e^AUC: area under curve.

**Table 4:** Metrics considering comprehensive geriatric assessment questionnaire subsets on decision tree (J48) classifier.

Metric	Subsets of questionnaires with occurrence						
	≥0 occurrences^a^	≥4 occurrences^b^		≥6 occurrences^c^		10 occurrences^d^	
	Mean (SD)	Mean (SD)	*P* value	Mean (SD)	*P* value	Mean (SD)	*P* value
Sensibility	70.34 (16.79)	75.16 (6.38)	.13	69.80 (12.13)	.47	62.12 (7.25)	.04
Specificity	62.89 (15.11)	71.67 (16.77)	.07	75.78 (26.22)	.11	84.56 (13.09)	.001
AUC^e^	67.55 (10.27)	78.79 (8.41)	.003	78.08 (8.74)	.006	76.97 (10.12)	.02

^a^≥0 occurrences: all comprehensive geriatric assessments (Charlson comorbidity index, geriatric depression scale-short form, international physical activity questionnaire-short form, Katz index of independence in activities of daily living, Karnofsky performance scale, mini-mental state examination, mini nutritional assessment-short form, polypharmacy, and timed up and go).

^b^≥4 occurrences: Charlson comorbidity index, geriatric depression scale-short form, Katz index of independence in activities of daily living, and mini nutritional assessment-short form.

^c^≥6 occurrences: Charlson comorbidity index and mini nutritional assessment-short form.

^d^10 occurrences: mini nutritional assessment-short form.

^e^AUC: area under curve.

**Table 5:** Metrics considering comprehensive geriatric assessment questionnaires subsets on multilayer perceptron classifier.

Metric	Subsets of questionnaires with occurrence				
	≥0 occurrences^a^	≥1 occurrence^b^		10 occurrences^c^	
	Mean (SD)	Mean (SD)	*P* value	Mean (SD)	*P* value
Sensibility	68.75 (8.34)	73.87 (9.68)	.03	77.41 (9.12)	.01
Specificity	62.67 (17.84)	74.89 (9.37)	.03	72.45 (12.35)	.03
AUC^e^	69.64 (9.83)	80.33 (6.86)	.005	82.33 (6.26)	.002

^a^≥0 occurrences: all comprehensive geriatric assessments (Charlson comorbidity index, geriatric depression scale-short form, international physical activity questionnaire-short form, Katz index of independence in activities of daily living, Karnofsky performance scale, mini-mental state examination, mini nutritional assessment-short form, polypharmacy, and timed up and go).

^b^≥1 occurrence: Karnofsky performance scale, mini-mental state examination, and mini nutritional assessment-short form.

^c^10 occurrences: Karnofsky performance scale and mini nutritional assessment-short form.

^e^AUC: area under curve.

## Discussion

### Principal Findings

Results indicate that the MNA-SF has greater predictive power to estimate the risk of early death in elderly cancer patients as it was selected on the 10-folds. MNA-SF is a rapid test validated for screening for nutritional risk and malnutrition in the elderly population. The predictive value of MNA-SF for early death may be related to the fact that the 6 MNA-SF questions cover areas other than just nutrition, which are frequently included in the CGA, such as mobility, neuropsychological disorders, and self-reported health, in addition to nutrition aspects, including weight loss, reduced food intake, and body mass index. In fact, low MNA-SF may reveal the effects of advanced disease in the overall health of patients, which also affects cancer-related mortality. A Brazilian study showed that abnormal nutritional status was an independent factor associated with hospital death among older patients with various chronic diseases, including cancer [34]. A similar association was also demonstrated in elderly Asian cancer patients who would receive first-line chemotherapy [35]. Finally, a French multicenter study with 348 elderly cancer patients aged 70 years and above also found that low MNA scores were associated with increased risk of premature death [36].

The results also indicated that KPS questionnaire has proven itself a valuable tool to estimate the risk of early death in elderly cancer patients. In the past decades, various studies have demonstrated the prognostic value of the KPS not only primarily for various cancers [37-40] but also for other disease entities [41]. It can also be considered as a significant indicator of hospitalization and survival time, in addition to identifying risk groups to assist in the orientation of patients to geriatric outpatients [42].

### Limitations

The efforts of this paper are a starting point. They provide solid evidences and some clinical recommendations. We proposed and developed simple ML models for the prediction of early death in elderly cancer patients. These models are accurate and precise and could be possibly used by clinicians to make proper treatment plans. However, additional research is needed to continue to strengthen the evidence base.

### Conclusions

The results showed that the MNA-SF and KPS have the highest predictive power to identify elderly patients at risk for early death. We strongly recommend that these questionnaires be incorporated into regular geriatric assessment of older patients with cancer.

The MNA-SF and the KPS requires only a few minutes to be completed. In addition, both can be easily managed by any member of the multidisciplinary team to help in the early identification of patients at risk, providing information that assists in the planning of interventions and improving the adherence to CGA in daily clinical oncology practice.

This study also has limitations that should be considered. This is a nonrandomized, single-center, exploratory study of a heterogeneous patient population similar to a real-life population of older patients with cancer. Conversely, some of its weaknesses could be considered the main strengths of the study: this is one of the few studies in Brazil that, in the clinical practice context of a Unified Health System oncology unit, investigated the use of ML algorithms in the prediction of early death in elderly cancer patients.

## Abbreviations

AUC: area under curve
CGA: Comprehensive Geriatric Assessment
CV: cross-validation
J48: Decision Tree
KPS: Karnofsky performance scale
ML: machine learning
MLP: multilayer perceptron
MNA-SF: Mini Nutritional Assessment-Short Form
NB: Naive Bayes
